# Motor congruency and multisensory integration jointly facilitate visual information processing before movement execution

**DOI:** 10.1007/s00221-019-05714-9

**Published:** 2020-02-08

**Authors:** J. A. Elshout, N. Van der Stoep, T. C. W. Nijboer, S. Van der Stigchel

**Affiliations:** 1 Experimental Psychology, Helmholtz Institute, Utrecht University, Utrecht, The Netherlands; 2 Center of Excellence for Rehabilitation Medicine, Brain Center Rudolf Magnus, University Medical Center Utrecht, Utrecht University and De Hoogstraat Rehabilitation, 3583 TM Utrecht, The Netherlands

**Keywords:** Eye movement, Hand movement, Attention, Motor congruency, Multisensory integration

## Abstract

Attention allows us to select important sensory information and enhances sensory information processing. Attention and our motor system are tightly coupled: attention is shifted to the target location before a goal-directed eye- or hand movement is executed. Congruent eye–hand movements to the same target can boost the effect of this pre-movement shift of attention. Moreover, visual information processing can be enhanced by, for example, auditory input presented in spatial and temporal proximity of visual input via multisensory integration (MSI). In this study, we investigated whether the combination of MSI and motor congruency can synergistically enhance visual information processing beyond what can be observed using motor congruency alone. Participants performed congruent eye- and hand movements during a 2-AFC visual discrimination task. The discrimination target was presented in the planning phase of the movements at the movement target location or a movement irrelevant location. Three conditions were compared: (1) a visual target without sound, (2) a visual target with sound spatially and temporally aligned (MSI) and (3) a visual target with sound temporally misaligned (no MSI). Performance was enhanced at the movement-relevant location when congruent motor actions and MSI coincide compared to the other conditions. Congruence in the motor system and MSI together therefore lead to enhanced sensory information processing beyond the effects of motor congruency alone, before a movement is executed. Such a synergy implies that the boost of attention previously observed for the independent factors is not at ceiling level, but can be increased even further when the right conditions are met.

## Introduction

The pre-motor theory of attention (PMT) postulates that attention is directed to a movement end location when a goal-directed motor action (e.g. an eye movement) is planned (Rizzolatti et al. 1987). Indeed, several studies have shown that spatial attention is shifted to the target location before an eye movement or reach movement is executed (Deubel and Schneider 1996; Hanning et al. 2018; Jonikaitis and Deubel 2011; Khan et al. 2011). These pre-movement shifts of attention are important to collect relevant information at the to-be-foveated/reached location and result in enhanced visual information processing of the movement target. Alternatively, Smith and Schenk (2012) suggest that activity in the motor system contributes to biased competition between sensory representations (Smith and Schenk 2012).

Whether different effectors of the motor system have independent resources for directing attention, is still under debate. Whereas some studies found that the eye movement system is dominant during visual guided reaching (Khan et al. 2011), other studies have shown that congruent eye- and reach movements to different targets can be executed with parallel distribution of attention to both targets (Hanning et al. 2018; Jonikaitis and Deubel 2011). In addition, combined eye- and hand movements executed to a common target result in a boost of attention towards that location, compared to the effects of a single movement (Hanning et al. 2018; Jonikaitis and Deubel 2011). Together, these studies suggest that, at least in specific situations, congruent motor actions can result in a larger pre-movement shift of attention than a motor action with a single effector.

The above-mentioned results were obtained using unisensory stimulation (e.g. only in the visual domain). However, in daily life we are, more often than not, exposed to multisensory events to which we make eye and hand movements to interact with our environment (think of, for example, the mouth movements and voice of a conversational partner when engaging in conversation or cars driving by in traffic situations). It is now well-known that the senses are intrinsically linked and that sensory information is integrated following certain principles, facilitating perception of the environment (Calvert et al. 2004; Spence and Driver 2004; van der Stoep et al. 2017). Multisensory stimuli are often reported to be more effectively processed (e.g. more accurate and precise localization, shorter response times and lower detection thresholds) than unisensory stimulation, when multisensory integration (MSI) occurs (Alais and Burr 2004; Ernst and Banks 2002; Hughes et al. 1994; Lovelace et al. 2003; Ross et al. 2007; Spence 2010; Stevenson et al. 2012; Van der Stoep et al. 2015). Two main principles that govern MSI are temporal (Chen and Vroomen 2013; Colonius and Diederich 2004; Frens et al. 1995; Meredith et al. 1987; Van der Stoep et al. 2015) and spatial proximity (Meredith and Stein 1986; Spence 2013; Stein and Stanford 2008; Stevenson et al. 2012). When, for example, sound and light originate from approximately the same spatial location (within the spatial binding window) and at approximately the same time (within the temporal binding window), it will be more likely that auditory and visual input will be integrated, resulting in facilitation of perception.

In conclusion, both congruence in the motor system and multisensory integration are factors that can facilitate visual information processing. Yet, whether both factors can work together and synergistically enhance visual information processing is not yet known. Such a synergy would imply that the boost of attention previously observed for the independent factors is not at ceiling level, but can be increased even further when the right conditions are met. In the current study we addressed these questions using multisensory stimuli (visual and auditory) in a 2-AFC visual discrimination task with short presentation times to specifically study motor congruency and MSI under pre-movement conditions.

## Methods

### Participants

Fifteen right-handed subjects (5 males, Mean age = 21.8 years, SD = 1.6) participated in this experiment. All subjects had normal or corrected-to-normal vision. Procedures were approved by the local ethical committee of the Faculty of Social Sciences at Utrecht University (FETC15-069). Four participants were excluded for further analysis based on the low number of remaining trials (< 15 trials for one of the conditions, see below for exclusion criteria).

### Setup

A similar setup as described in Deubel et al. (1998) was used to present the stimuli (Deubel et al. 1998). In this setup participants were instructed to make combined eye- and hand movements to a target presented at an eccentricity of 10° from fixation at the centre of the screen. The target was presented on the left or right side of the screen and surrounded by 2 distractors on each side (spaced 3° visual angle). Before movement execution, the target (“E” or “3”) is masked and participants report whether an “E” or “3” was presented (2-AFC).

Participants sat in a dark room with their chin resting on a chinrest, and their right hand placed beneath a one-way mirror on a raised surface tilting towards them. This mirror reflects light from above and allows light to pass through from underneath. Hence while in a darkened room, participants were unable to see their hand below. Stimuli were presented on a computer screen positioned above this mirror, visible to participants in the reflection at 57 cm distance. An LED light clipped onto the end of the right index finger illuminated after finishing the reach movement and was visible through the mirror. This light provides participants with visual feedback on the end position of the reach, for them to gage the accuracy of their movement. Reach movements were recorded using a MiniBird (Ascension Technologies) with a resolution of 100 Hz. Eye movements were recorded by an Eyelink 1000 (for 5 participants) or Eyelink II system (SR Research Ltd. Ottawa ON; sampling rate of 1000/250 Hz., respectively). The Eyelink II system resulted in less data loss caused by interfering hand movements. Participants used their left hand to make a response, by pressing either the left or right arrow key on a keyboard, positioned on the table to their left. They kept two fingers resting upon these keys throughout the experiment, ready to make a response.

### Procedure

**Fig. 1:**
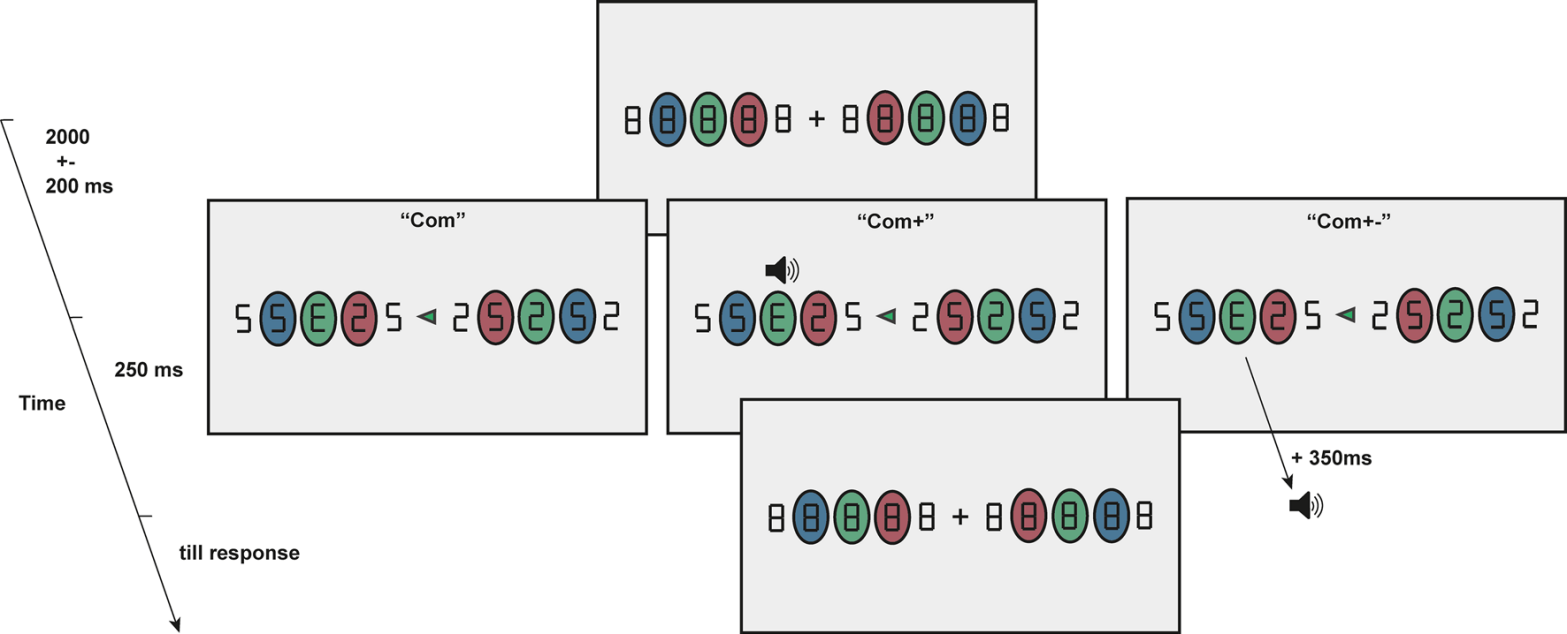
Visual discrimination task. Participants make combined eye and hand movements to the green target locations (10° eccentricity). Note that the stimuli (“8”, “5”, “2”, “E’”, and “3”) were white in the actual experiment, but adjusted to black in this figure for clarity

A simplified version of the visual discrimination task by Deubel and Schneider (1996) was used, similar to that described previously (Khan et al. 2009). Participants maintained fixation on a central fixation cross. On each side of the fixation cross, five white (100% luminance screen, i.e. 300 cd/m^2^) visual targets (figure “8” symbols, spaced 3°) were presented on a grey background (50% luminance screen) of which the middle three were surrounded by coloured ovals (Fig. 1). The participants were instructed to always make the congruent eye and hand movement to the figure in the center of these five figures (spaced 3° visual angle), surrounded by the green oval at an eccentricity of 10° from fixation at the center of the screen (left or right). The instruction to make a movement to the left or right was indicated by the appearance of a central green arrow cue pointing to the left or right and presented after a random interval between 1800 and 2200 ms after fixation onset. Movement side was randomized and counterbalanced. As soon as the movement cue appeared, participants had to make the simultaneous eye and hand movement to the figure in the green oval. At the same time of the cue, all figures “8” were replaced by a figure “2” or “5” for a period of 250 ms after which the figures were masked again by the figures “8”. One of the figures located at a position surrounded by an oval was replaced by an “E” or “3” and the participant had to respond (forced choice) whether an “E” or “3” was presented by pressing the right or left arrow key on the keyboard, respectively. This target figure was always at the same side as the movements, and 60% of the trials at the movement location (green oval) and 40% of the trials on a movement irrelevant location (20% red oval and 20% blue oval).

Each participant was tested in three different conditions: (1) visual target without any sound (“Com”), (2) visual target with sound temporally and spatially aligned (“Com+ ”) and (3) visual target with sound temporally misaligned (350 ms after the onset of the visual stimulus) and spatially aligned (“Com+ −”). The sound was a white noise burst presented via 2 stereo speakers placed beneath the mirror on the raised and tilted surface [~ 70 dB(A)].

Each participant first practiced with saccade only and reach only movements to get familiar with the task. For each experimental condition, we collected 240 trials per participant (two blocks of condition “Com” and four blocks of condition “Com+ ” and “Com+ −” intermixed).

Prior to the experiment, each participant performed a short panning task to match the subjective perceived sound location to the location of the green oval (10° of eccentricity to the left and right from the center of the screen). To this end, we presented a 60 dB white noise burst at ten panning values between zero (only left speaker active) and one (only right speaker active). The participant indicated with a mouse click the location on the screen he/she thought the sound is coming from.

### Data analysis

Saccade and reach movements were analysed offline. Trials were excluded if one of the movements (1) was to the wrong side, (2) landed on the target before masking (latency + duration < 250 ms), or (3) had an amplitude < 2° visual angle. Based on these criteria, further analysis was performed for eleven participants. A two-way repeated measures ANOVA between condition (com, com+ and com+ −) and location (movement target, movement irrelevant) was used to analyse the performance.

## Results

For eleven participants, at least ~ 50% of all trials in each condition remained. This percentage is not very surprising given the difficulty of the task and is comparable to the amount of data loss reported by Khan et al (2011). Of note, the same analysis as described below with all data lead to similar results with the same conclusion.

### Movement onset latencies

**Fig. 2:**
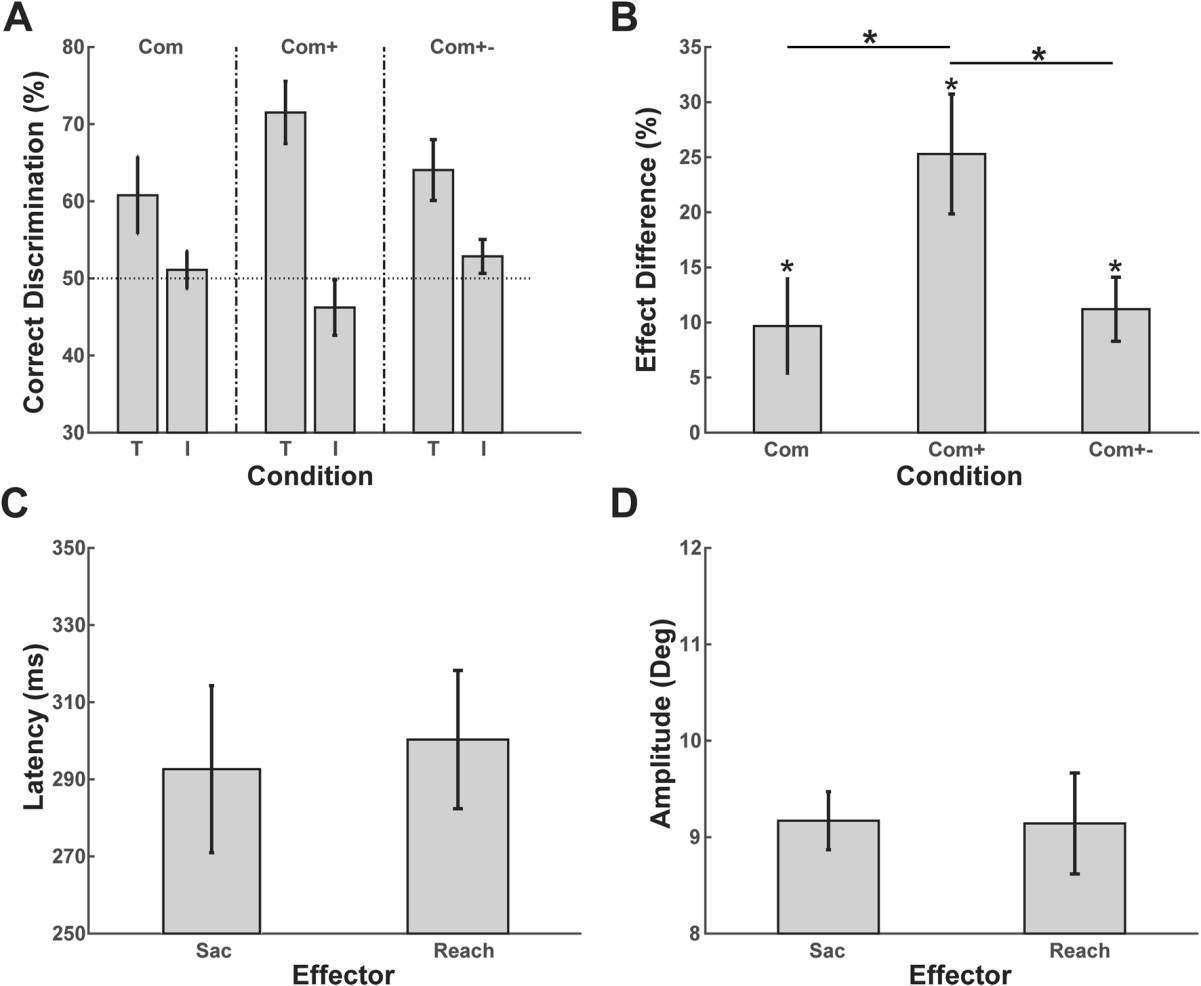
**a** Performance during combined eye and hand movements without sound (Com), with sound spatially and temporally aligned (Com+) and with sound temporally misaligned (Com+ −). *T* movement target location; *I* movement irrelevant location. The dotted line at 50% reflects chance level. **b** Difference scores for each condition. **c** Mean latencies (±SE). **d** Mean amplitudes (±SE)

The mean saccade and reach latencies across all conditions (Fig. 2c) were 293 ms (SE = 21.65 ms) and 300 ms (SE = 17.92 ms), respectively. In the Com condition (combined movements, no sound) the saccade latency was 305 ms (SE = 24.87 ms) and the reach latency was 292 ms (SE = 17.30 ms). In the Com+ condition (sound temporally aligned), the saccade latency was 272 ms (SE = 17.47 ms) and the reach latency was 283 ms (SE = 20.20 ms). In the Com+− condition (sound temporally misaligned) the saccade latency was 303 ms (SE = 25.29 ms) and the reach latency was 325 ms (SE = 20.54 ms). A repeated-measures ANOVA indicated that saccade latencies differed between conditions [F(2, 20) = 3.898, *p* = 0.037, $\eta_{{\text{p}}}^{2}$ = 0.28]. However, post hoc pairwise comparisons between all conditions suggest no difference between them (all *t* < 2.205; all *p* > 0.051, uncorrected).

### Movement amplitude and response times

The mean saccade and reach amplitudes across all conditions were 9.17° (SE = 0.31°) and 9.14° (SE = 0.52°), respectively (Fig. 2d). The saccade amplitudes per condition (Com, Com+ and Com+ −) were 9.31° (SE = 0.46°), 9.09° (SE = 0.27°) and 9.10° (SE = 0.29°), respectively. A repeated-measures ANOVA with a Greenhouse–Geisser correction determined that we have no reason to assume that the mean saccade amplitudes differed between conditions [*F*(1.115, 11.149) = 0.307, *p* = 0.6151].

The reach amplitudes per condition (Com, Com+ and Com+ −) were 9.34° (SE = 0.57°), 9.08° (SE = 0.58°) and 9.01° (SE = 0.62°), respectively. A repeated-measures ANOVA with a Greenhouse–Geisser correction did not show any significant main effects. Therefore, we have no reason to assume that the mean reach amplitudes differ between conditions [*F*(1.143, 11.427) = 0.29, *p* = 0.631].

The mean manual response time across all conditions (answer to the 2AFC task) was 714 ms (SE = 100.29 ms). The response times per condition (Com, Com+ and Com+ −) were 734 ms (SE = 116.1 ms), 680 ms (SE = 95.9 ms) and 728 ms (SE = 93.4 ms), respectively. A repeated-measures ANOVA with a Greenhouse–Geisser correction determined that we have no reason to assume that the mean response time differ between conditions [*F*(1.079, 10.789) = 1.427, *p* = 0.261].

### Visual detection performance (2AFC task)

A two-way repeated-measures ANOVA revealed an interaction between condition (Com, Com+ and Com+ −) and location (movement target, movement irrelevant) *F*(2, 20) = 5.442, *p* = 0.013, $\eta_{{\text{p}}}^{2}$ = 0.352 (Fig. 2a). There was a main effect of location *F*(1, 10) = 16, *p* = 0.003, $\eta_{{\text{p}}}^{2}$ = 0.615. There was no main effect of condition *F*(2, 20) = 0.648, *p* = 0.534, $\eta_{{\text{p}}}^{2}$ = 0.061. Overall, performance was higher for movement target locations (*M* = 65.45, SE = 1.22) than for movement irrelevant locations (*M* = 50.06, SE = 0.29). The interaction indicates that the difference in performance between the movement target and movement irrelevant location varied between conditions. Therefore, we calculated this difference score for each condition by subtracting the performance on the movement irrelevant location from the movement relevant location. Planned pairwise comparisons between conditions using *t* tests revealed that the performance in the MSI condition (Com+) was higher than both the combined condition (Com) and the combined condition with sound temporally misaligned (Com+ −, *t* = − 2.579, *p* = 0.027 and *t* = 2.462, *p* = 0.034, respectively, Fig. 2b). The difference score of all conditions was different from 0 (all *t* > 2.007; all *p* < 0.036, one-tailed), indicating that attention was shifted to the target locations prior to movement execution in all conditions.

Together our findings show that congruence in the motor system and MSI together lead to enhanced sensory information processing before a movement is executed.

## Discussion

In the current study we investigated whether congruence in the motor system and MSI can jointly enhance visual information processing before movement execution. While previous studies have shown that both factors individually facilitate visual information processing (Hanning et al. 2018; Jonikaitis and Deubel 2011; Lovelace et al. 2003; Stein and Stanford 2008; Van der Stoep et al. 2015), this is the first study to demonstrate that MSI and motor congruency can jointly facilitate visual information processing before the execution of a combined hand–eye movement. Such a synergy implies that the boost of attention previously observed for the independent factors is not at ceiling level, but can be increased even further when the right conditions are met.

More specifically, we compared a unisensory visual condition (Com), and two multisensory (audiovisual) conditions: one in which visual and auditory stimuli were temporally aligned (Com+) and one in which they were not and the visual stimuli always preceded the auditory stimulus (Com+ −). Previous studies have reported that multisensory stimulation (e.g. visual + auditory) can enhance visual spatial attention (Spence 2010). In line with the principle of temporal alignment, we only observed facilitation in the Com+ condition in which the auditory and visual stimuli were presented temporally aligned. When the visual and auditory stimuli were temporally misaligned (Com+ −) performance was similar to the condition with only motor congruency (Com). Theoretically, only in the condition with spatially and temporally aligned visual and auditory stimuli (Com+), MSI can occur. Given that we presented the auditory stimulus within the temporal binding window in the Com+ condition to allow MSI (Chen and Vroomen 2013; Spence and Squire 2003), the enhanced visual information processing in this condition is therefore likely the result of a combination of a pre-motor shift of attention and MSI.

Note that we compared the condition with motor congruence + MSI (Com+) to the condition with motor congruency (Com) without MSI, and not a MSI only condition without the motor congruency effect. Studying MSI alone without movement execution in our setup is not possible as any increased spatial attention at the action endpoint in this condition could also be the result of congruence in motor plans and not execution per se: according to the PMT, planning of a movement is sufficient to direct attention (Rizzolatti et al. 1987). It is impossible to know when participants planned but did not execute eye and/or hand movements. For this reason, we only selected trials with accurate execution to confirm accurate planning. However, to be sure that our data are not the result of an unintended selection bias based on the criteria applied for motor execution, we performed the same analysis again, but this time included all subjects and all trials. This analysis lead to qualitatively similar results with the same conclusions.

The fact that we find enhanced visual information processing when motor congruency and MSI co-occur (compared to motor congruency alone) suggests that both processes jointly contribute to enhanced visual information processing. Several options come to mind when thinking about the underlying neuronal processes responsible for the additive effects of motor congruency and MSI. For example, both processes might be integrated in a specific brain area involved in both motor congruency and MSI. Alternatively, motor congruency and MSI might contribute separately at different levels of processing, similarly resulting in the observed additive effect of facilitation of visual information processing.

If we speculate about the neural networks involved in motor congruency effects, findings by Jonikaitis and Deubel (2011) suggest that both effectors of the motor system have separate attentional mechanisms that are integrated at later processing stages. In line with their findings, activity in different parietal cortex regions was found for preparatory eye and hand movements in fMRI and MEG studies (Tosoni et al. 2008; Van Der Werf et al. 2010). It has been proposed that the lateral intraparietal part (LIP) of the posterior parietal cortex contains an effector-independent saliency map which can be used to direct attention to important spatial locations (Fecteau and Munoz 2006; Goldberg et al. 2006). The LIP is therefore an important area involved in directing attention during combined eye and hand movements. Interestingly, LIP is also known to be involved in multisensory integration (Ghazanfar and Schroeder 2006). The traditional view in MSI research states that each unisensory component of a multisensory stimulus is initially processed independently and integrated at a later stage of processing (Treisman and Gelade 1980). However, this view is being challenged since studies have found compelling evidence of multisensory integration in early sensory processing areas that were long thought to be solely unisensory (for review, see (Ghazanfar and Schroeder 2006). Together, we speculate that LIP might be the key area where congruence in the motor system and multisensory integration both facilitate sensory information processing.

To conclude, this is the first study that shows that congruency in the motor system and MSI can synergistically enhance visual information processing compared to congruence in the motor system alone. A combination of both factors can be of direct relevance for rehabilitation programs in patient groups with difficulties in perception, such as stroke patients suffering from visuospatial neglect. Whereas visual scanning training (learning to make systematic eye movements to the affected hemifield) is now the golden standard to treat visuospatial neglect, an adapted version of scanning training with congruence in the motor system (i.e. eye + hand movements) and multisensory stimulation (allowing for MSI), might be of great value for the rehabilitation process.
